# Male Goltz Syndrome due to Postzygotic *PORCN* Mosaicism: A Case Report

**DOI:** 10.1155/crdm/1302419

**Published:** 2026-09-15

**Authors:** Vu Anh Dao Tran, Mohammed Eldoadoa, Tong Ba Tran, Nhi Thi Uyen Pham, Lam D. Chau, Abdelrahman M. Makram, Nguyen Tien Huy, Thuy Thi Phan Nguyen, Randa Elsheikh

**Affiliations:** ^1^ Department of Dermato-Venereology, Ho Chi Minh City Hospital of Dermato-Venereology, Ho Chi Minh City, Vietnam; ^2^ Department of Orthopaedics, St Helens & Knowsley NHS Teaching Hospital Trust, Merseyside, UK; ^3^ Department of Biochemistry, Case Western Reserve University, Cleveland, Ohio, USA, case.edu; ^4^ School of Public Health, Imperial College London, London, UK, imperial.ac.uk; ^5^ Institute of Research and Development, Duy Tan University, Da Nang, Vietnam, duytan.edu.vn; ^6^ School of Medicine and Pharmacy, Duy Tan University, Da Nang, Vietnam, duytan.edu.vn; ^7^ School of Tropical Medicine and Global Health (TMGH), Nagasaki University, 1-12-4 Sakamoto, Nagasaki 852-8523, Japan, nagasaki-u.ac.jp; ^8^ Deanery of Biomedical Sciences at Edinburgh Medical School, University of Edinburgh, Edinburgh, UK, ed.ac.uk

**Keywords:** focal dermal hypoplasia, Goltz syndrome, Goltz–Gorlin syndrome, *PORCN* variant

## Abstract

Goltz syndrome, also known as focal dermal hypoplasia (FDH), is a rare X‐linked dominant genetic disorder caused by loss‐of‐function mutations in the *PORCN* gene, which is crucial for Wnt protein secretion and signaling during embryonic development. The syndrome primarily affects females, as hemizygous males with pathogenic variants in the gene typically die in utero, while surviving males are usually mosaic for the variant. Patients typically present with diverse phenotypic features, including dermal hypoplasia, musculoskeletal abnormalities, ocular malformations, and dental defects, often distributed asymmetrically along Blaschko’s lines. Despite the identification of the *PORCN* gene as the main gene involved in the pathogenesis of the disease, a significant gap remains in understanding the broad spectrum of *PORCN* mutations and the resulting variability in clinical presentation among Goltz syndrome patients, particularly concerning the factors that contribute to the differences in disease severity and expression. This study reports a 9‐year‐old male patient presenting with non‐healing skin lesions, syndactyly, limb length discrepancy, and scoliosis, consistent with clinical findings of Goltz syndrome. Whole‐exome sequencing identified a hemizygous nonsense variant in the *PORCN* gene (c.915G > A), resulting in a truncated protein (p.Trp305Ter). This nucleotide‐level variant is distinct from, but affects the same residue as, a previously reported pathogenic variant reported by Lasocki et al., further supporting the clinical relevance of truncating changes at this codon and providing insight into the mosaicism associated with milder male phenotypes.

## 1. Introduction

Goltz syndrome (OMIM #305600), also known as Goltz–Gorlin syndrome or focal dermal hypoplasia (FDH), is a rare mesoectodermal X‐linked dominant genetic disorder [1] that was first described in 1962 [2]. It is caused by loss‐of‐function mutations in the *PORCN* gene, the human homolog of Drosophila’s porcupine protein, located on the X chromosome (Xp11.23) [3]. The gene is responsible for the secretion and signaling of Wnt proteins, which are involved in the embryological interaction between the mesoderm and ectoderm [4]. The syndrome mainly affects females; hemizygous males with gene variants are usually not viable in utero, and surviving males are typically mosaic [4].

Goltz syndrome may affect any part of the body in an asymmetric pattern and is characterized by dermal hypoplasia, in correspondence with Blaschko’s lines, with fat often protruding through dermal gaps. This is frequently accompanied by musculoskeletal defects, such as syndactyly, brachydactyly, and ectrodactyly, and ocular malformations such as coloboma, enophthalmos, microphthalmia, and cataract. Additionally, craniofacial asymmetry, dental abnormalities, and malformations in the gastrointestinal and central nervous system have been described [1, 4].

The variant spectrum of *PORCN* is diverse, with over 200 variants classified as pathogenic or likely pathogenic currently documented in the Leiden Open Variation Database (LOVD) and ClinVar (as of February 15, 2026). Among these reported variants, nonsense variants represent the most prevalent class of molecular alterations (https://databases.lovd.nl/shared/genes/PORCN). Although somatic mosaicism has been proposed as a mechanism permitting survival in affected males, the genetic and phenotypic spectrum of Goltz syndrome remains poorly understood.

We hereby report a male patient with mosaic Goltz syndrome in whom a hemizygous postzygotic nonsense variant of the *PORCN* gene was identified.

## 2. Case Presentation

**FIGURE 1:**
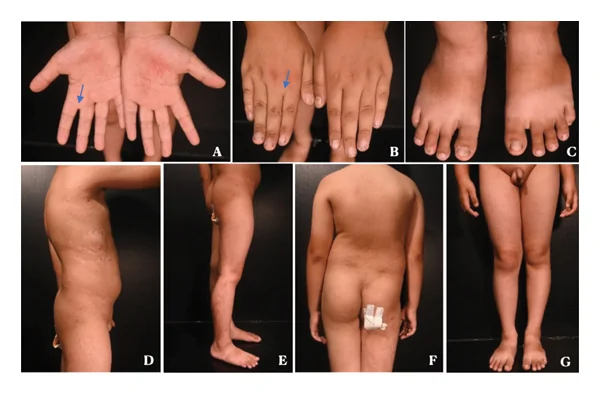
Clinical features exhibited by our case: (A, B) Cutaneous syndactyly of the 2nd and 3rd fingers on the right hand (blue arrows); (C) ridged nails; (D, E). atrophic skin with nodular fat herniation clustered on the right‐side trunk and leg; (F) scoliosis; (G) limb length discrepancy.

A 9‐year‐old male with multiple skin abnormalities was referred to the Department of Cosmetics at the Dermato‐Venereological Hospital of Ho Chi Minh City, Vietnam. The patient complained of several non‐healing skin lesions, distributed over his trunk and lower limbs. Upon examination, hypopigmented aplastic patchy areas and scars were observed over the right side of his body, in correspondence with the distribution of Blaschko lines. Additionally, nodular fat herniation clustered on the right side of his trunk was noticed. Partial syndactyly of the right index and middle fingers was also present, with ridges over the nails of the right thumb and right third toe. Furthermore, scoliosis, limb length discrepancy, and inguinal hernia were observed (Figure 1). A diagnosis of Goltz syndrome was suspected clinically. Importantly, family history did not reveal any record of genetic diseases.

**FIGURE 2:**
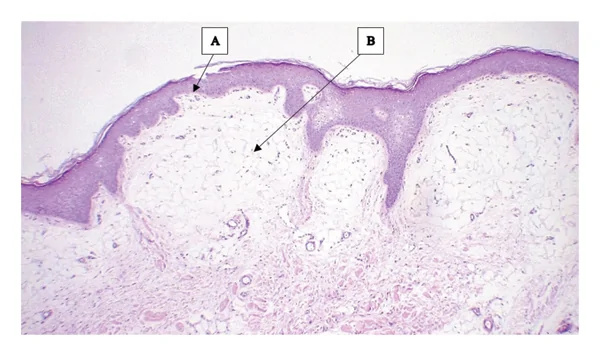
Histopathological section of the skin (H&E, ×10). The epidermis appears relatively preserved (A), while the dermis is markedly thinned and shows replacement by lobules of mature adipose tissue extending abnormally close to the epidermis (B), consistent with focal dermal hypoplasia.

Skin biopsies were performed from the atrophic patches, which showed a microscopic picture of normal epidermis, markedly reduced dermal thickness, loosely arranged collagen bundles, and adipose tissue replacement in the papillary dermis. Adipocytes could be seen extending to the undersurface of the epidermis (Figure 2).

To confirm the diagnosis, blood samples were obtained, and whole‐exome sequencing (WES) was performed to identify variants in the *PORCN* gene. Genomic DNA was extracted from peripheral blood and affected skin tissue of the proband using standard protocols. Genomic DNA from a normal male control was also included for comparison in Sanger sequencing. WES was performed on genomic DNA from the proband following our previously established next‐generation sequencing workflow, as described by Bui et al. [5]. Briefly, exonic regions and flanking splice‐site sequences were captured using a commercial exome enrichment platform, followed by paired‐end sequencing on an Illumina sequencing platform. Raw sequencing reads were assessed for quality and aligned to the human reference genome. Variants were called using a standard bioinformatics pipeline and annotated according to gene location, predicted protein consequence, population frequency, disease association, and clinical relevance. Candidate variants were prioritized based on the patient’s clinical phenotype, inheritance pattern, predicted functional effect, and absence or rarity in population databases. Particular attention was given to variants in genes associated with FDH and X‐linked disorders. At the *PORCN* locus, WES identified the variant, NM_203475.3:c.915G > A, predicted to result in a premature termination codon, p.Trp305Ter. Manual review of the aligned reads showed a total sequencing depth of 97 reads at the variant position, including 7 alternate allele reads and 90 reference allele reads. The estimated variant allele fraction was therefore 7.2%/VAF = 7/97, supporting a low‐level mosaic variant. Visual inspection of the aligned reads was performed using Integrative Genomics Viewer (IGV), which showed a small cluster of alternate allele‐supporting reads against a predominant reference‐read background, consistent with mosaicism.

Sanger sequencing was performed as an orthogonal validation method. Sanger sequencing was performed on DNA extracted from the proband’s affected skin and peripheral blood, as well as DNA from a normal male control. The chromatograms were compared across samples. The normal male control showed only the reference allele, whereas the proband showed mosaic alternate peaks in both skin and blood samples. These Sanger results confirmed the NGS finding and supported the presence of *PORCN* mosaicism in the male proband. The pathogenicity of the variant was interpreted according to the American College of Medical Genetics and Genomics/Association for Molecular Pathology guidelines. The *PORCN* c.915G > A; p.Trp305Ter variant was classified as pathogenic based on PVS1, PM2, and PP4 criteria: It is a predicted loss‐of‐function variant in a gene where loss of function is a known disease mechanism, it is absent or extremely rare in population databases, and the patient’s phenotype is highly consistent with *PORCN*‐related FDH.

Multidisciplinary supportive management was initiated: conservative wound care and emollient therapy for the nonhealing skin lesions; a height‐increasing insole for limb length discrepancy; and scheduled inguinal hernia repair. The family was counseled regarding future surgical correction of scoliosis, syndactyly repair, and laser therapy for hypopigmentation.

Genetic sequencing was not performed on the patient’s parents. Given the postzygotic de novo nature of the variant, the recurrence risk for the parents is not expected to be increased. However, the patient may carry a risk of transmitting the variant to his daughters. The family was counseled accordingly and advised to seek formal genetic counseling in the context of future reproductive planning.

## 3. Discussion

This study describes a male patient with mosaic Goltz syndrome with a hemizygous nonsense *PORCN* variant. The most plausible mechanism in this male patient is a postzygotic de novo *PORCN* variant resulting in somatic mosaicism. This explanation is consistent with the low‐level alternate allele signal observed by NGS (variant allele fraction 7.2%, sequencing depth 97×) and Sanger confirmation in both skin and blood. Because hemizygous nonmosaic pathogenic *PORCN* variants are usually embryonically lethal in males, surviving affected males are typically mosaic. Novel hemizygous *PORCN* mutations have been reported by Frisk et al. [3] and Alkindi et al. [6]. Similar to our patient, patchy skin lesions and syndactyly were also observed in the reported patients, which, upon genetic testing, revealed previously unreported mosaic variants of the disease [3, 6].

Other symptoms have also been reported in the literature, including dental defects, ocular abnormalities, cardiac defects, pulmonary hypertension, renal anomalies, and diaphragmatic hernia [3]. In 2016, Bostwick et al. proposed diagnostic criteria for Goltz syndrome based on skin and limb findings. They suggested that a diagnosis of Goltz syndrome should be suspected in patients with at least three of the following skin findings: congenital patchy skin aplasia, congenital nodular fat herniation, congenital hyper/hypopigmentation following Blaschko linear distribution, telangiectasia, or congenital ridged nails. These findings should be coupled with at least one of the following limb defects: ectrodactyly, transverse limb defects, oligodactyly, syndactyly, or significant long bone reduction [7].

Nonetheless, despite the high frequency of skin and musculoskeletal presentations, cases of Goltz syndrome without skin defects have been reported [8–10], indicating that genetic testing—using methods capable of detecting low‐level mosaicism—should be considered in all clinically suspected cases, including those without overt skin manifestations.

Several pathogenic variants have been associated with Goltz syndrome. Although the disease primarily affects females, male patients have also been reported [11–16], which are thought to be caused by de novo postzygotic variants occurring in the early developmental stage. Affected male patients are typically mosaic; it is this mosaicism, rather than selective lethality alone, that allows for the possibility of father‐to‐daughter transmission [10]. Interestingly, de novo mosaic variants are associated with milder phenotypic findings [1], which might explain why some affected parents might not be detected until the birth of a child with overt Goltz syndrome [10].

While X‐chromosome inactivation and skewing occur in females with *PORCN* point mutations and deletions, these mechanisms have not been directly linked to phenotypic severity. Instead, clinical variability is more strongly correlated with the degree of postzygotic mosaicism, particularly in male patients [6].

In this patient, the low‐level allele fraction (7.2%) is inconsistent with inheritance of the variant on a single hemizygous X chromosome, which would be expected to yield a near‐complete variant allele fraction; instead, it supports a postzygotic mutational event in which only a subset of cell lineages carries the variant while the remaining cells retain a wild‐type X chromosome, accounting for the coexistence of wild‐type and variant alleles without invoking Klinefelter syndrome (47, XXY) or parental mosaicism. Consistent with a de novo origin, there was no family history of Goltz syndrome or related features, and both parents were clinically unaffected; however, this could not be molecularly confirmed, as parental genetic testing was not performed. Although Klinefelter syndrome is not required to explain the molecular findings above, it was not formally excluded by karyotype analysis in our patient, which represents an acknowledged limitation, particularly given that Klinefelter syndrome has been reported in association with Goltz syndrome in male patients and could theoretically constitute an alternative mechanism for male survival with an X‐linked pathogenic variant. Notably, a pathogenic variant affecting the same protein residue (p.Trp305Ter) has been previously reported [17], further supporting the clinical relevance of truncating variants at this position.

Goltz syndrome may be confused with similar conditions such as aplasia cutis, MIDAS (microphthalmia, dermal aplasia, and sclerocornea), nevus lipomatosus superficialis, and microphthalmia with linear skin defects (MLS) [1]. Skin biopsy of the hypoplastic area is considered the current gold standard for the diagnosis of the condition as it shows the characteristic picture of decreased dermal thickness, loosely arranged collagen bundles, and penetration of the adipose tissue in the dermis and epidermis. Treatment of Goltz syndrome is mainly supportive and requires a multidisciplinary team composed of ophthalmologists, dermatologists, pediatricians, general surgeons, plastic surgeons, and orthopedic surgeons [15].

Several limitations of this study should be acknowledged. First, parental genetic testing was not performed; however, given the postzygotic de novo mechanism, this would not have been expected to substantially alter the genetic counseling provided. Furthermore, even WES would have limited ability to detect very low‐level parental mosaicism. The estimated variant allele fraction of 7.2% falls at the lower threshold of standard WES sensitivity for somatic mosaicism; Sanger sequencing therefore served as an important orthogonal validation, confirming the variant in both blood and affected skin.

Our study describes a patient with Goltz syndrome in which a postzygotic mosaic variant was discovered. This variant adds to the spectrum of known pathogenic variants in the *PORCN* gene, providing further insight into the genetic basis and mechanisms of this syndrome. Given the rarity of the condition, continued research on *PORCN* gene variants and related pathways is essential to better understand the pathogenesis and develop effective treatments for Goltz syndrome.

## Author Contributions

Vu Anh Dao Tran: conceptualization, clinical investigation, data curation, and writing–original draft. Mohammed Eldoadoa: formal analysis, writing–original draft, and writing–review and editing. Tong Ba Tran: clinical investigation and resources. Nhi Thi Uyen Pham: clinical investigation and data curation. Lam D. Chau: formal analysis, methodology (genetic analysis), and writing–review and editing. Abdelrahman M. Makram: formal analysis, writing–original draft, and writing–review and editing. Nguyen Tien Huy: supervision and writing–review and editing. Thuy Thi Phan Nguyen: conceptualization, supervision, resources, and writing–review and editing. Randa Elsheikh: conceptualization, methodology, project administration, supervision, writing–original draft, and writing–review and editing.

## Funding

No funding was received for the conduction of this study.

## Ethics Statement

Our institution does not require ethical approval for case reports. Hence, ethical approval for this study was waived.

Written informed consent, including consent for images publication, was obtained from the child’s parents.

## Consent

Written informed consent was obtained from the child’s parents.

## Conflicts of Interest

The authors declare no conflicts of interest.

## Supporting Information

Additional supporting information can be found online in the Supporting Information section.

## Supporting information


**Supporting Information** Supporting Figure 1. (A‐B). IGV snapshot views of the *PORCN* locus on chromosome X in a normal male control (A) and the proband patient (B), showing reference‐only reads in the control and low‐level alternate reads (arrow) in the proband, consistent with mosaicism. (C) Sanger sequencing confirmed the NGS result in the patient’s skin and blood samples, with mosaic alternate peaks detected in skin tissues (arrow), while the normal male control showed only the reference peak. Standard WES may have limited sensitivity for detecting low‐level mosaic variants; Sanger sequencing was therefore performed as an orthogonal validation method to confirm the NGS finding.

## Data Availability

The raw data are available for the journal to review.
