# Supplementary material for: Male Goltz Syndrome due to Postzygotic PORCN Mosaicism: A Case Report
Source: Case Rep Dermatol Med. 2026 Sep 15;2026:1302419. doi: 10.1155/crdm/1302419 (PMC13575781; doi:10.1155/crdm/1302419)
Supplement: Supplementary file 1 [file CRDM-2026-1302419-s001.pdf]

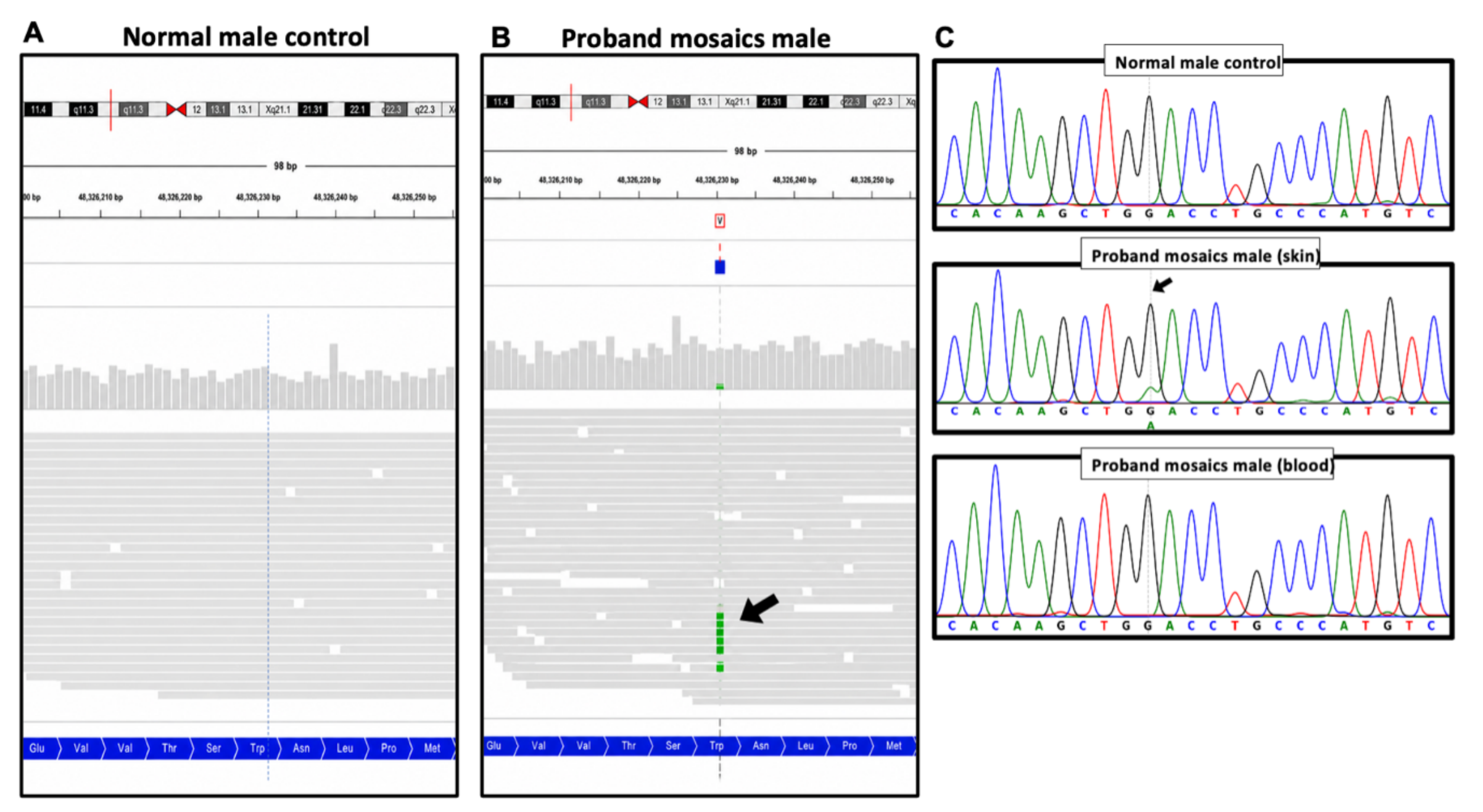

A
Normal male control
B
Proband mosaics male
C
Normal male control
Proband mosaics male (skin)
Proband mosaics male (blood)
98 bp
Glu Val Val Thr Ser Trp Asn Leu Pro Met
C A C A A G C T G G A C C T G C C C A T G T C
